# Similar temperature dependencies of glycolytic enzymes: an evolutionary adaptation to temperature dynamics?

**DOI:** 10.1186/1752-0509-6-151

**Published:** 2012-12-07

**Authors:** Ana Luisa B Cruz, Marit Hebly, Giang-Huong Duong, Sebastian A Wahl, Jack T Pronk, Joseph J Heijnen, Pascale Daran-Lapujade, Walter M van Gulik

**Affiliations:** 1 Department of Biotechnology, Delft University of Technology and Kluyver Centre for Genomics of Industrial Fermentation, Julianalaan 67, Delft, 2628 BC, The Netherlands; 2 Netherlands Consortium for Systems Biology, PO Box 94215, Amsterdam, 1090 GE, The Netherlands

**Keywords:** Glycolysis, Kinetic modelling, Metabolomics, *Saccharomyces cerevisiae*, Temperature dynamics

## Abstract

**Background:**

Temperature strongly affects microbial growth, and many microorganisms have to deal with temperature fluctuations in their natural environment. To understand regulation strategies that underlie microbial temperature responses and adaptation, we studied glycolytic pathway kinetics in *Saccharomyces cerevisiae* during temperature changes.

**Results:**

*Saccharomyces cerevisiae* was grown under different temperature regimes and glucose availability conditions. These included glucose-excess batch cultures at different temperatures and glucose-limited chemostat cultures, subjected to fast linear temperature shifts and circadian sinoidal temperature cycles. An observed temperature-independent relation between intracellular levels of glycolytic metabolites and residual glucose concentration for all experimental conditions revealed that it is the substrate availability rather than temperature that determines intracellular metabolite profiles. This observation corresponded with predictions generated *in silico* with a kinetic model of yeast glycolysis, when the catalytic capacities of all glycolytic enzymes were set to share the same normalized temperature dependency.

**Conclusions:**

From an evolutionary perspective, such similar temperature dependencies allow cells to adapt more rapidly to temperature changes, because they result in minimal perturbations of intracellular metabolite levels, thus circumventing the need for extensive modification of enzyme levels.

## Background

Growth and survival of microorganisms is strongly affected by environmental variables such as temperature, nutrient and oxygen availability, pH and osmolarity. Since, in natural environments, these parameters are highly dynamic, microorganisms have to cope with fluctuating, often non-optimal growth conditions. Suboptimal growth temperatures have major impacts on cell physiology including decreasing membrane fluidity and a reduced efficiency of protein synthesis and folding [1-3]. In addition, the catalytic capacity of each enzyme in the cell decreases when the temperature is lowered. This temperature impact can, in many cases, be described by an Arrhenius equation [4].

In the past decade, the response of the mesophilic yeast *Saccharomyces cerevisiae* to suboptimal temperatures has been the focus of several studies [5-7]. Interest in this subject is motivated by the biotechnological applications of *S. cerevisiae*. In particular, brewing and winemaking are two processes in which yeast is subjected to suboptimal temperatures (typically 12 to 15°C) to obtain specific desired flavour compounds [5,8]. Moreover, its experimental accessibility to genome-scale analysis makes *S. cerevisiae* an attractive model organism for systems biology studies on temperature responses. With a few exceptions [2,9], studies on low temperature responses of *S. cerevisiae* have focussed on so-called cold shock experiments. In such experiments, instantaneous exposure to low temperatures triggers a general environmental stress response in addition to temperature-specific responses [2,9-12]. To investigate long-term acclimation rather than rapid adaptation to low temperature, thereby preventing a cold shock effect, growth of *S. cerevisiae* has been studied at 30 and 12°C in anaerobic glucose-limited chemostat cultures [6]. Since the maximum specific growth rate of *S. cerevisiae* at 12°C is circa sevenfold lower than at 30°C [6,13], a low dilution rate of 0.03 h^-1^ was used for both temperatures in this chemostat study [5,6,14]. In anaerobic cultures, substrate-level phosphorylation in glycolysis is the sole mechanism for ATP synthesis. Tai *et al.*[6] observed that, despite substantially lower specific catalytic capacities of the glycolytic enzymes at 12°C as compared to 30°C, chemostat cultures maintained the same glycolytic flux at these two temperatures. Because in these chemostat cultivations the growth rate was set to be the same for both conditions (0.03 h^-1^), this indicated that the biomass yield was the same for both temperatures. Moreover, yeast did not compensate for the lower temperature by increased synthesis of glycolytic enzymes. Instead, metabolic regulation, i.e., regulation by changes in the concentrations of substrates, products and effectors [15] was identified as the main strategy for temperature compensation. Especially for highly expressed pathways such as glycolysis, whose enzymes can account for up to 20% of the protein content of *S. cerevisiae*, repeated cycles of protein degradation and synthesis would represent a substantial burden. It was therefore hypothesized that the observed dominant role of metabolic regulation represents an evolutionary adaptation to environments with frequent (e.g. circadian) temperature fluctuations [6].

Nevertheless, physiological studies carried out at tightly controlled constant temperatures or during very fast temperature changes (e.g. cold or heat shock experiments) represent artificial conditions, considering that many microorganisms are exposed to circadian and seasonal temperature fluctuations in their natural habitats. Evolution in such habitats is likely to have resulted in regulatory strategies to optimize performance under dynamic temperature regimes. Analysing and understanding such strategies is a typical systems biology challenge, and requires integration of biological experiments with mathematical modelling [16-18].

**Figure 1:**
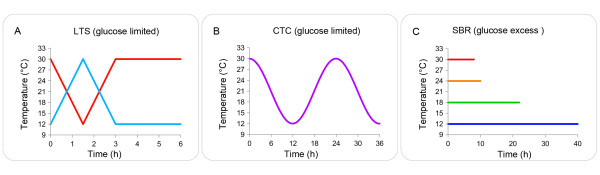
**Temperature profiles applied in the different simulations and experiments.** (**A**) linear temperature shifts (LTS) from 12°C (blue) or 30°C (red) applied to steady-state chemostats; (**B**) circadian temperature cycles (CTC) in glucose-limited chemostat cultures; (**C**) Sequential batch reactors (SBR) operated at different temperatures.

The aim of this study is to identify and understand mechanisms employed by *S. cerevisiae* to control glycolytic flux and intracellular metabolite levels under dynamic temperature regimes. To this end, we investigated the impact of dynamic temperature regimes with different time constants (Figure 1) using a systems approach, integrating mathematical modelling and experimentation. Our results indicate that if the temperature dependencies of the catalytic capacities of enzymes in a pathway are highly similar, changes in metabolite levels during temperature changes are minimal.

## Results

### A minimal model to describe temperature dependency of metabolic fluxes

To understand and model the impact of temperature dynamics on metabolic fluxes, it is essential to consider the influence of temperature on the kinetic parameters of enzyme catalyzed reactions. The *in vivo* rate of an enzyme-catalyzed reaction depends on the concentration of the enzyme (*e*), its specific catalytic capacity (*k*_*cat*_), the affinities of the enzyme for substrates, products, co-factors and/or inhibitors (usually represented by the saturation constants *K*_*P*_, *K*_*S*_, or *K*_*E*_) and the thermodynamic equilibrium constant (*K*_*eq*_) (equation (1)).

(1) $$v=e.k_{\mathrm{cat}}.f\left(K_{P},K_{S},K_{E},S,P,E\right).\left(1-\frac{\Gamma }{K_{\mathrm{eq}}}\right)$$

In this rate equation, the specific format of the mechanistic kinetic function *f(K*_*P*_*, K*_*S*_*, K*_*E*_*, S, P, E)* depends on the catalytic mechanism of the reaction. The mass-action ratio (Γ) only depends on the stoichiometry and thermodynamic properties of the reaction [19].

Three main questions need to be addressed when modeling the impact of temperature on metabolic networks:

1. Which kinetic parameters are temperature dependent?

2. How can temperature dependency be described for those who are?

3. Can the same mechanism be applied to all enzymes?

It is well known that each enzyme has an optimum temperature at which its catalytic capacity (*k*_*cat*_) is maximal, while *k*_*cat*_ decreases at values below and above the optimum temperature (for a review see [3]). Quantitative relations, describing the impact of temperature on *k*_*cat*_*,* can be established by *in vitro* activity measurements at different temperatures and at saturating reactant concentrations. There are, however, only few data available on *in vitro* enzyme activity measurements at different temperatures for yeast glycolytic enzymes. A recent study focused on only two different temperatures (12 and 30°C), thus precluding the derivation of a proper quantitative relation for the temperature dependency of *k*_*cat*_[6].

Even when information about *in vitro* temperature-dependencies of enzymes was available, this would not necessarily provide an accurate reflection of the *in vivo* situation [20]. Analysis of the temperature impact on enzyme levels (*e*) needs to be done carefully, since these are intrinsically context dependent. For example, glycolytic enzyme levels in *S. cerevisiae* are strongly influenced by specific growth rate [21], nutrient limitation regimes [22] and number of generations [23]. Tai *et al.*[6] minimized these influences by growing *S. cerevisiae* in anaerobic chemostat cultures at 12 and 30°C under otherwise identical conditions. Under these conditions, the glycolytic flux was the same for both temperatures and the levels of glycolytic enzymes were found to be very similar (average absolute fold change 1.5 ± 0.1) at both temperatures. In the same experiments, *k*_*cat*_, estimated from *in vitro* enzyme assays, was 3.9 ± 0.5 fold lower at 12°C than at 30°C. It was therefore inferred that changes in enzyme levels are not the primary regulation mechanism used by cells to, at equal flux, compensate for the loss of catalytic capacity at lower temperatures. Similar conclusions were drawn by Postmus *et al.*[14], when studying the impact of supra-optimal temperatures on the regulation of glycolytic flux in aerobically grown *S. cerevisiae*.

Changes in temperature might also affect the equilibrium constants of reactions and the binding affinities of enzymes. The temperature impact on the equilibrium constant *K*_*eq*_ is described by the Van’t Hoff equation (equation (2)). When applying this equation, it can be found that the changes of the equilibrium constants for the glycolytic reactions differ by less than twofold (in average change 1.2 ± 0.2) for temperatures between 12 and 30°C (Table 1).

(2) $$\ln\left(\frac{K_{eq,2}}{K_{eq,1}}\right)=\frac{\Delta H_{r}^{0}}{R}\left(\frac{1}{T_{1}}-\frac{1}{T_{2}}\right)$$

**Table 1:** **Thermodynamic equilibrium constants (K**_**eq**_**) of selected glycolytic reactions at 12 and 30°C**

**Enzyme**	**Abrev.**	**K**_**eq**_		**Fold change**
		**12°C**	**30°C**	
Hexokinase	HXK	7.7 x10^3^	3.5 x10^3^	0.45
Phosphoglucose isomerase	PGI	0.27	0.29	−0.93
Phosphofructokinase	PFK	5.0 x10^3^	2.4 x10^3^	2.08
Fructose-1,6-biphosphate aldolase	FBA	9.3 x10^-4^	1.4 x10^-3^	−1.51
Triosphosphate isomerase	TPI	0.040	0.048	−1.20
Glyceraldehyde-3-phosphate dehydrogenase	TDH	0.66	0.69	−1.05
Phosphoglycerate kinase	PGK	20	16	1.25
Phosphoglycerate mutase	PGM	0.074	0.087	−1.18
Enolase	ENO	5.1	4.5	1.13
Pyruvate kinase	PYK	3.9 x10^4^	1.4 x10^4^	2.79

K_eq_ values were calculated from [24] considering a reference condition of 298.15 K, *I* = 0.25M and pH 7.0. The fold change refers to the ratio K_eq_^ 12°C^/K_eq_^ 30°C^.

Based on a mechanistic description [25], it can be assumed that temperature has a minor impact on the binding affinities of the enzymes, because they are a measure of the equilibrium between the enzyme and the enzyme-substrate complex. Furthermore, temperature changes within the mesophilic range (10 to 40°C) do not result in major structural alterations of the active sites of enzymes from mesophilic microorganisms [26,27]. Therefore, changes in binding affinities caused by temperature-induced structural changes are expected to be negligible. In the mesophilic range, temperature induced changes in the glycolytic flux are therefore mainly caused by changes of *k*_*cat*_, because this effect is by far the largest and works in the same direction for all enzymes. Consequently, the temperature sensitivity of *k*_*cat*_ for the different enzymes in a network will determine its overall response to dynamic temperature conditions. This raises the key question whether the catalytic capacities of all enzymes of a pathway would have different or identical temperature dependencies. Consider for instance the following simple linear pathway where *A* and *B* are intracellular metabolites, while *S* and *P* are extracellular:

(3) $$S\overset{v_{1}}{\to }A\overset{v_{2}}{\to }B\overset{v_{3}}{\to }P$$

Under steady-state conditions (no accumulation of metabolites) all fluxes are the same, i.e. v_1_ = v_2_ = v_3_. This can be formally written by a system of equations (4), representing the steady-state balances of intracellular metabolites *A* and *B*, where *N* is the matrix containing the stoichiometric coefficients of the reactions.

(4) $$\left[\begin{array}{ccc} \begin{array}{l} 1\\ 0 \end{array} & \begin{array}{l} -1\\ 1 \end{array} & \begin{array}{l} 0\\ -1 \end{array} \end{array}\right]\cdot \left[\begin{array}{c} v_{1}\\ v_{2}\\ v_{3} \end{array}\right]=\left[\begin{array}{c} 0\\ 0 \end{array}\right]↔\text{N}\cdot \left[\begin{array}{c} v_{1}\\ v_{2}\\ v_{3} \end{array}\right]=0$$

Taking into account that the fluxes *v*_*i*_ can be described by rate equations of the form of equation (1), the system of two metabolite balances becomes, for a reference temperature (T_0_):

(5) $$N\cdot \left[\begin{array}{c} e_{1}\cdot k_{cat,1}^{T_{0}}\cdot f\left(K_{A},S,A,K_{eq,1}\right)\\ e_{2}\cdot k_{cat,2}^{T_{0}}\cdot f\left(K_{A},K_{B},A,B,K_{eq,2}\right)\\ e_{3}\cdot k_{cat,3}^{T_{0}}\cdot f\left(K_{B},B,K_{eq,3}\right) \end{array}\right]=0$$

When it is furthermore assumed that the pathway is irreversible (as, for instance, anaerobic yeast glycolysis), the concentration of *P* plays no role. This set of algebraic relations can then be solved to obtain the intracellular metabolite levels at the reference temperature, for given values of the extracellular concentration of *S*, thus yielding relations for the intracellular levels of *A* and *B* as a function of the extracellular concentration of substrate *S* for the reference temperature. It should be noted here that this conclusion is also valid for pathways containing reversible reactions, as long as the steady-state assumption is fulfilled.

Assuming that the catalytic capacity of each enzyme as a function of temperature can be described by the function *R*_*i*_*(T)* and the rate at the reference temperature, such that ${k_{cat,}}_{i}=k_{cat,i}^{T_{0}}.R_{i}\left(T\right)$ and that the stoichiometry does not change with temperature [13], the description of the system at a temperature *T* different from *T*_*0*_ becomes:

(6) $$N\cdot \left[\begin{array}{c} e_{1}\cdot R_{1}\left(T\right)\cdot k_{cat,1}^{T_{0}}\cdot f\left(K_{A},S,A,K_{eq,1}\right)\\ e_{2}\cdot R_{2}\left(T\right)\cdot k_{cat,2}^{T_{0}}\cdot f\left(K_{A},K_{B},A,B,K_{eq,2}\right)\\ e_{3}\cdot R_{3}\left(T\right)\cdot k_{cat,3}^{T_{0}}\cdot f\left(K_{B},B,K_{eq,3}\right) \end{array}\right]=0$$

If each enzyme has its own temperature dependent function *R*_*i*_*(T)*, the ratio between *A*, *B* and *S* will be different for each temperature and dependent on the parameters of the corresponding temperature function. If, on the other hand, the temperature function of the single enzymes follow the same mechanism with the same parameters, e.g. *R*_*1*_*(T) = R*_*2*_*(T) = R*_*3*_*(T)*, then eq. (6) will become equal to eq. (5). This would then imply that the relations describing the intracellular concentrations of *A* and *B* as a function of the extracellular substrate concentration are temperature independent. The differences between these two scenarios can be tested *in vivo* to provide insight into the temperature dependency of enzymes.

Considering the simple network described above, we now assume a dynamic temperature situation in which the concentration of *S* is high and non-limiting and thus fluxes *v*_*1*_ to *v*_*3*_ are at their maximum values. If the catalytic capacities of the enzymes in a pathway or network share the same temperature dependency, changes in temperature will cause all enzyme activities to change with the same factor. In such a scenario, no changes in intracellular metabolite levels will occur and thus the cells maintain complete homeostasis during temperature changes, without the need to adjust enzyme levels. If, however, for a substrate-limited system (i.e. the flux through the pathway is limited by the supply of *S*) it is experimentally imposed that the fluxes remain the same even at lower temperature (e.g. in a chemostat cultures at constant dilution rate), *S* as well as the metabolite concentrations *A* and *B* will change to compensate for the lower enzymatic capacities.

### *In silico* evaluation of different *k*_*cat*_ -temperature relationships in yeast glycolysis

To evaluate the impact of different or identical temperature dependencies of the *k*_*cat*_ of the enzymes in yeast glycolysis, simulations were carried out with a detailed kinetic model of this pathway developed by Teusink *et al.*[20]. Several modifications were made to the published version of the model to, amongst others, account for biomass growth and to include temperature dependency of enzyme capacities (see Materials and Methods). Using this model different substrate feeding regimes and temperature profiles were simulated to distinguish between the effects of temperature and of extracellular glucose concentration on glycolytic flux. This distinction is especially important for glucose-limited conditions, where the glycolytic flux is highly correlated with the extracellular glucose concentration because, as shown by [28] and [29], glucose transport is a key growth-rate-controlling process. Therefore, in glucose-limited chemostat cultures, temperature dynamics will affect the extracellular glucose concentration via changes in glycolytic capacity. These changes in the extracellular glucose concentration will then propagate through all intracellular metabolite levels, yielding temperature-independent relations between intracellular metabolite and extracellular glucose levels. If, on the other hand, a different relationship between intracellular metabolite levels and extracellular glucose concentrations is observed for different temperature profiles, this would indicate that the temperature impact on *k*_*cat*_ differs for the different enzymes in yeast glycolysis (see simple example in the preceding paragraph).

Two distinct rounds of simulations with different substrate feeding regimes were performed to evaluate the impact of the parameters of the temperature-dependent function *R*_*i*_*(T)* on the intracellular metabolite profiles. In the first round, the temperature sensitivity of one of the glycolytic enzymes was set to be different from all others. In the second round, temperature sensitivities were set to be identical for all enzymes, implying that a decrease in temperature resulted in the same relative decrease of *k*_*cat*_ for all enzymes. In both rounds, simulations were performed for circadian temperature cycles, linear temperature shifts starting from 30°C steady-state conditions and batch cultures grown at 12, 18, 24 and 30°C (temperature profiles are illustrated in Figure 1).

**Figure 2:**
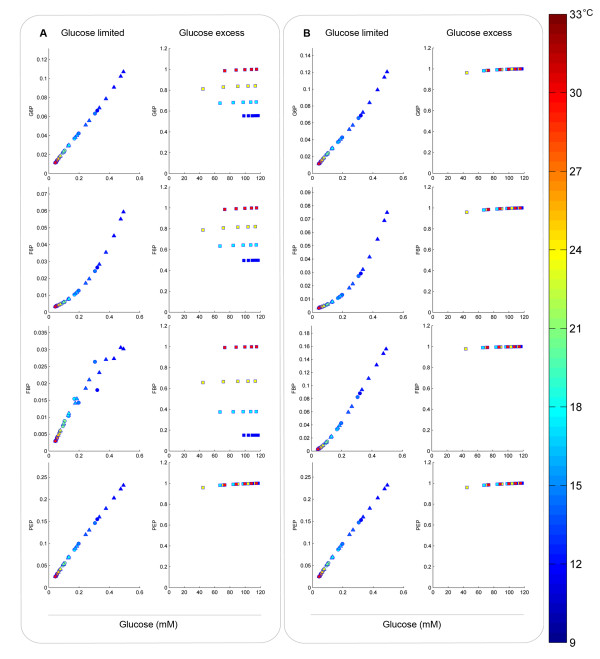
**Simulation results of intracellular metabolite levels, normalized to the levels under glucose-excess conditions at 30°C.** (**A**) *k*_*cat*_ of glyceraldehyde 3-phosphate dehydrogenase (TDH) was set to be 2 times less sensitive to temperature than the other glycolytic enzymes; (**B**) all *k*_*cat*_ were set to have the same temperature dependency. The symbols refer to simulations of: sinoidal temperature cycles (△), linear temperature shifts applied to 30°C steady-state chemostats (○) and batch fermentations at different temperatures (□). The colors indicate the culture temperature at the time of sampling. Simulation results considering different temperature sensitivities for phosphofructokinase and pyruvate kinase can be found in the Additional file 1: Figures S1A and B.

All simulations resulted in trends between intracellular metabolite levels and the extracellular glucose concentration. As expected, different temperature profiles or substrate-feeding regimes caused different trends when at least one of the enzymes was set to have a different temperature sensitivity compared to the others. For instance, when the temperature sensitivity of the *k*_*cat*_ of glyceraldehyde 3-phosphate dehydrogenase (TDH) was set to be two fold lower than that of the other glycolytic enzymes, the simulated values of the intracellular G6P, F6P and FBP levels under glucose-excess conditions clearly decreased with decreasing temperature (Figure 2A). Differences were less pronounced for the model simulations of glucose-limited conditions, where metabolites upstream of TDH (G6P, F6P) showed no differences and only FBP was mildly affected by the change in temperature sensitivity. Furthermore FBP levels correlated poorly with the extracellular glucose concentration, especially for the fastest temperature shifts applied (circles in Figure 2A). Similar results were obtained when changing the sensitivity of the other glycolytic enzymes (see Additional file 1: Figures S1A and B), indicating that this observation is not related to the flux towards the reaction, nor to its kinetic mechanism or reversibility. It was noticed that only metabolites upstream of the reaction with the different temperature sensitivity displayed such deviating trends. These observations are consistent with experimental and modeling results of [30] on the impact of changes in the abundance of single enzymes in metabolic pathways and with the ‘passive network mechanism’ proposed by these authors.

When temperature sensitivities were assumed to be identical for all glycolytic enzymes, the intracellular concentrations of glycolytic intermediates under glucose-excess conditions were predicted to be temperature independent (Figure 2B). Conversely, simulations of glucose-limited conditions revealed strong changes of intracellular metabolite concentrations during the temperature shifts. The range of intracellular levels was broader than in the previous simulation (Figure 2A). Nevertheless, because the temperature sensitivity was set to be identical for all enzymes, intracellular metabolite concentrations showed highly similar correlations with the extracellular glucose concentration, irrespective of the simulated temperature profile. The results of these simulations agree with the results obtained with the simple example network (equation 3), thus showing that model predictions are independent of the assumed kinetic mechanisms of the enzymes involved, as long as the assumption of temperature independent *K*_*i*_ holds true. Therefore, experimental analysis of the relations between the extracellular glucose concentration and intracellular metabolite levels for different cultivation conditions and temperature regimes should resolve the question whether or not all enzymes in a pathway share the same *k*_*cat*_ -temperature relationship. Although based on model simulations the major differences between the two hypotheses are expected in glucose-excess conditions, *in vivo* there might be more than one enzyme with significantly different temperature dependency. Therefore, the deviating trends of intracellular metabolite levels simulated in glucose-limited conditions compared to glucose-excess conditions might be augmented. Experimental analysis under both conditions is important to decipher and quantify the temperature dependency of the glycolytic enzymes.

### Experimental evaluation of model predictions

To experimentally investigate *k*_*cat*_ -temperature relationships in yeast glycolysis, anaerobic cultures were grown under different substrate feeding regimes and dynamic temperature conditions. The experimental setups used included batch cultures at 12, 18, 24 and 30°C, to evaluate the temperature impact on intracellular metabolite levels under glucose excess conditions (sequential batch experiments (SBR)) and different sets of dynamic temperature shifts between 12 and 30°C performed in glucose-limited chemostat cultures. These dynamic experiments comprised short term (3 h) linear temperature shifts (12→30→12°C) and (30→12→30°C) applied to steady-state cultures at 12 and 30°C (LTS12 and LTS30) and sinoidal circadian temperature cycles (CTC) with 24 h frequency (Figure 1). Experiments LTS12 and LTS30 aimed at understanding if different pre-cultivation temperatures would affect the metabolic response to temperature shifts. The different timescale of the temperature perturbations between LTS and CTC allowed to better discriminate between impact of temperature and substrate concentration (see previous paragraphs). The CO_2_ production rate was monitored on-line during all experiments since, in anaerobic cultures, it provides an accurate measure for (changes in) glycolytic flux.

**Figure 3:**
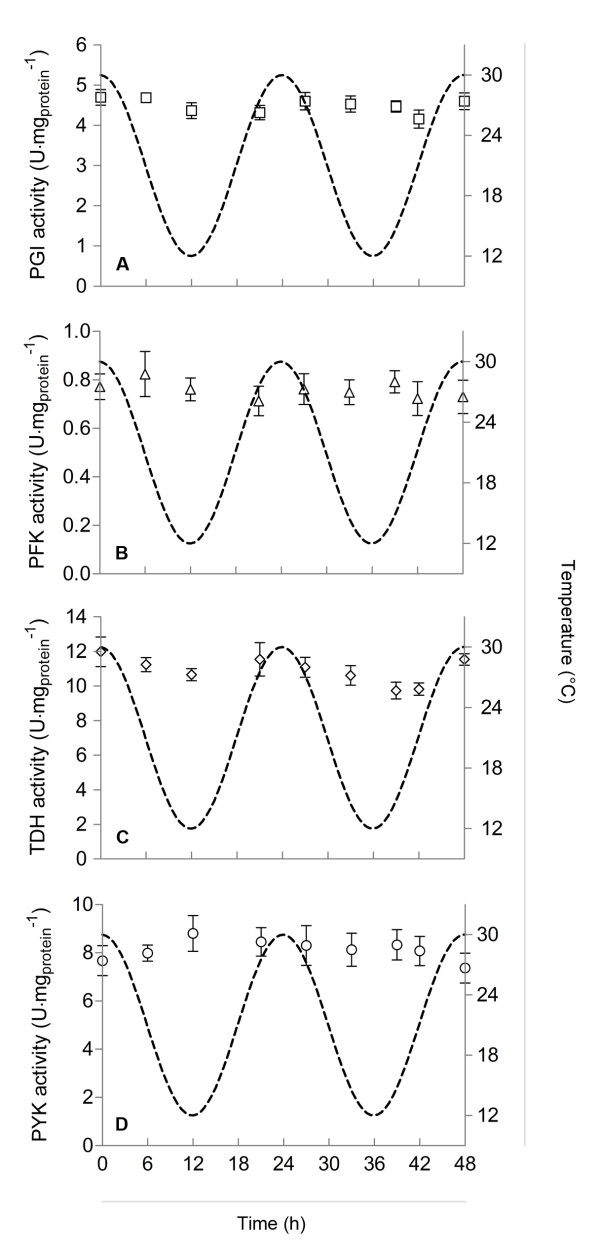
**Measured biomass specific capacities (*V***_max_**) of four glycolytic enzymes, during circadian temperature cycles in glucose limited chemostats.***V*_max_ values were obtained from *in vitro* enzyme activity assays performed at 30°C in cell free extracts. A. Phosphoglucose isomerase (PGI); B. Phosphofructokinase (PFK); C. Glyceraldehyde-3-phosphate dehydrogenase (TDH); D. Pyruvate kinase (PYK). Results for the other glycolytic enzymes can be found in the Additional file 2 .

**Figure 4:**
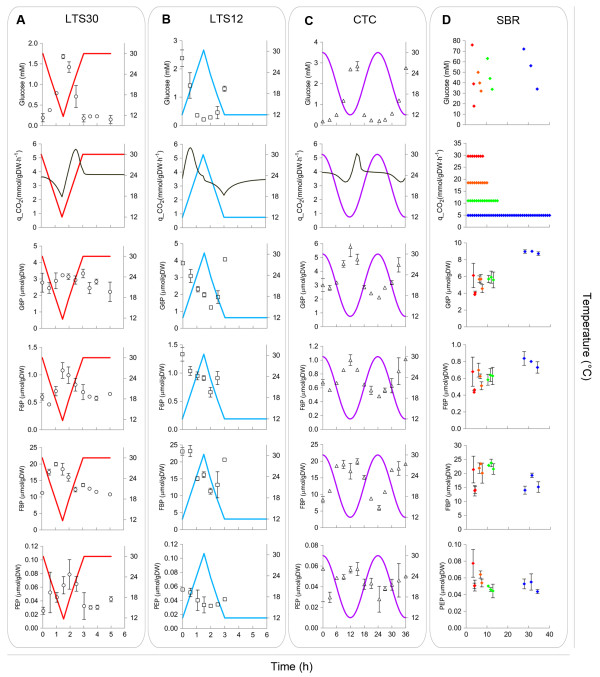
**Experimental results of residual glucose concentration, CO**_**2**_**production and intracellular glycolytic metabolite levels versus time for fast linear temperature shifts (A and B), circadian temperature cycles (C) and batch cultivations (D) at different temperatures.** The colored solid lines in panels A, B and C represent the applied temperature profiles. Error bars indicate the standard error of the average from at least two duplicate samples and two independent cultures.

*In vitro* enzyme activity assays were performed during CTC cultivation, since this setup had the slowest temperature dynamics and, consequently, the highest chance of hierarchical regulation of the glycolytic flux (i.e., changes in enzyme levels). Nevertheless, levels of glycolytic enzymes did not significantly change during the temperature cycles (Figure 3 and Additional file 2). This observation, together with previous results [6], confirms that temperature changes have negligible impact on glycolytic enzyme levels in *S. cerevisiae*. On the other hand, intracellular metabolite levels and extracellular glucose concentrations showed a dynamic behavior in the CTC and LTS experiments (Figure 4).

In the SBR experiments, glucose was present in excess, and therefore the glycolytic flux depended exclusively on the cultivation temperature, through its impact on *k*_*cat*_. Even though the glycolytic flux in batch cultures was observed to be six-fold lower at 12°C than at 30°C, intracellular metabolite levels were independent of the growth temperature (Figure 4D).

**Figure 5:**
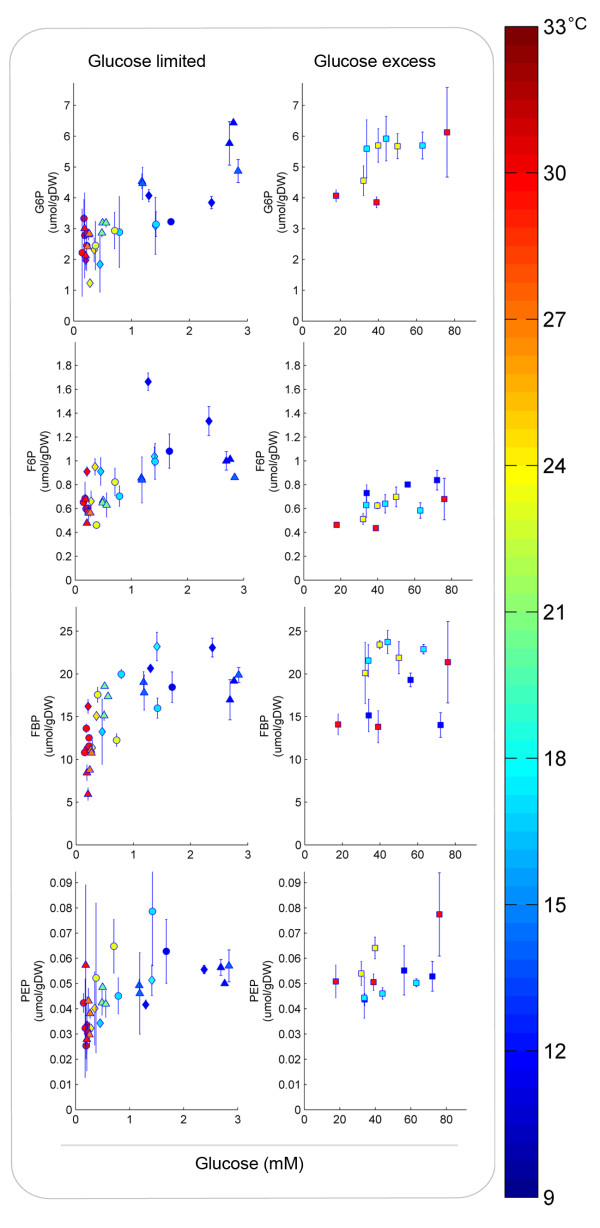
**Experimentally obtained relations between intracellular levels of glycolytic intermediates and extracellular glucose concentration.** The different symbols represent data from different experiments, i.e. sinoidal temperature cycles (△), linear temperature shifts starting at 30°C (○) or 12°C (⋄) and batch cultivations at different temperatures (□). Error bars refer to the standard error of the average of at least two duplicate samples and two independent runs of experiments. Colors indicate the temperature of the culture at the time of sampling.

When temperature dynamics were applied to anaerobic glucose-limited chemostat cultures (LTS and CTC experiments), the residual glucose concentration was observed to increase with decreasing temperature as response to a decrease of the glucose transport capacity. As a result, the metabolite profiles show the inverse dynamics when compared to the temperature profiles applied to the chemostat cultivations (Figure 4A-C). When the intracellular metabolite levels, measured during the LTS and CTC experiments, were plotted in one graph as a function of the residual glucose concentration, a single relation between metabolite level and residual glucose was obtained (Figure 5 and Additional file 3). It can be inferred from these results that the metabolic response was independent of the initial steady-state temperature (12 or 30°C) or the dynamics of the temperature perturbation applied, indicating that hierarchical regulation of yeast glycolytic flux does not play a significant role during temperature dynamics. Furthermore, based on the *in silico* evaluation of different scenarios, these results support the notion that all glycolytic enzymes in *S. cerevisiae* share a similar temperature-*k*_*cat*_ relationship.

## Discussion

Considering that chemical reactions in aqueous solutions have activation energies between 42 and 125 kJ.mol^-1^, it can be estimated that reaction rates change by two fold for every 10°C temperature change [31]. However, temperature effects on individual enzyme-catalysed reactions can deviate substantially from this ‘rule of thumb’, for example due to the impact of temperature on protein structure. As illustrated with a simple linear metabolic pathway (equation 3), different temperature dependencies of enzyme reactions inevitably result in (large) changes in concentrations of pathway metabolites. Similar changes in metabolite levels are observed when expression levels of individual enzymes in a pathway are modified [30]. Hierarchical regulation, i.e. regulation at the level of enzyme synthesis [15], would then be needed to restore and maintain homeostasis.

Previous *in vitro* assays in cell extracts, performed at 12 and 30°C, suggested large differences in temperature dependency for the ten glycolytic enzymes in *S. cerevisiae*[6]. However, *in vitro* studies with optimized assays, in which parameters such as pH and concentrations of salts, cofactors and effectors differ for each enzyme, are not representative for the intracellular environment [32]. The differences in, among others, protein content, osmotic pressure, substrate diffusion between the *in vitro* and *in vivo* conditions might lead to different flux versus temperature relationships.

**Figure 6:**
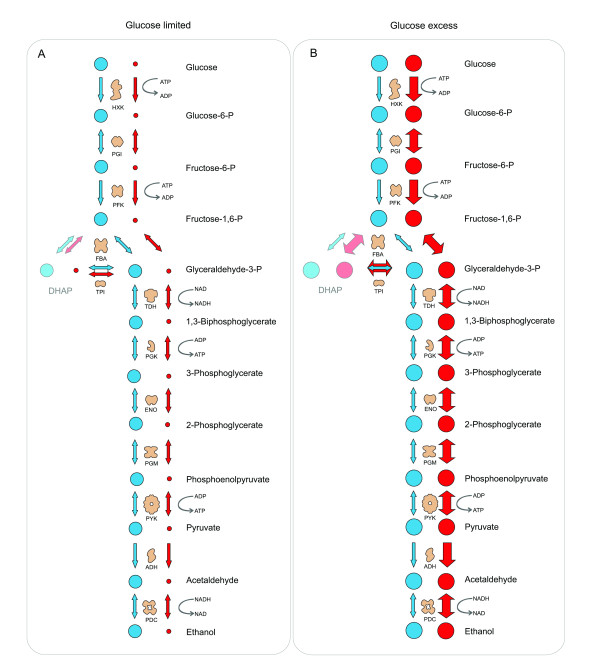
**Overview of the impact of temperature on glycolytic flux in anaerobic cultures of *S. cerevisiae.*** The two panels represent growth under glucose-limited (A) and glucose-excess (B) conditions. The thickness of the arrows and diameter of the circles reflect the relative flux through the glycolytic enzymes and the intracellular concentrations of glycolytic intermediates, respectively. Blue and red symbols indicate growth at 12°C and 30°C, respectively.

To avoid the inherent problems of *in vitro* studies on enzyme kinetics, we developed a novel systems biology approach for analyzing kinetic regulation strategies under temperature dynamics. By combining *in silico* simulation of different strategies with experimental analysis of metabolite and substrate concentrations under various controlled temperature and substrate-feeding regimes, regulation strategies could, for the first time, be analyzed without relying on kinetic parameters derived from *in vitro* experiments. The *in silico* simulations predicted that similar temperature dependencies of the *k*_*cat*_ of all glycolytic enzymes should result in a single relation between metabolite level and residual substrate concentration for each metabolite, independent of temperature. Consequently, in batch cultures growing at saturating substrate concentrations, intracellular concentrations of glycolytic intermediates should be independent of the growth temperature. In glucose-limited chemostat cultures, each metabolite level is then predicted to solely depend on the residual glucose concentration and not on the temperature as such (Figure 6).

Experimental results obtained under conditions of glucose excess, were consistent with this scenario and could not be reconciled with the alternative scenario in which temperature dependencies of the *k*_*cat*_ values of individual glycolytic enzymes were different. Although the experimental results of metabolite levels versus residual glucose for the glucose limited chemostat experiments (LTS and CTC) showed similar trends as the model simulations, the experimental errors of the metabolite data did not allow discriminating between the two simulated scenarios (i.e. the same temperature dependency for all enzymes versus a single enzyme different). However, if several glycolytic enzymes have different temperature sensitivities, the differences should be larger. Unfortunately, simulations in which several glycolytic enzymes have different temperature sensitivities did not result in a stable model.

All simulations were carried out using the model parameters published in [20]. It should be realized, however, that different initial conditions, in our case anaerobic glucose-limited chemostat cultures at 12 and 30°C, could have resulted in different expression levels of glycolytic isoenzymes, which could have led to differences in enzyme kinetic properties in both conditions. A genome wide transcriptome analysis of cells cultivated under both conditions [6] did not provide indications for significant differences in the expression of glycolytic isoenzymes. Nevertheless, changes were observed in the expression levels of the different hexose transporters between the 12 and 30°C chemostat cultivations. Determination of the glucose transport kinetics for both conditions indeed showed that the glucose transport capacity of cells grown in 12°C chemostats was higher than that of cells grown at 30°C [6]. Changes in the kinetics of glucose transport could have had a repercussion on the metabolite profiles as a function of the residual glucose concentration between shifts LTS12 and LTS30. Nevertheless, it was measured that the increase in the *k*_*cat*_ of glucose transport at 12°C was coupled to an increase in the glucose saturation constant (*K*_*S*_), in such a way that the ratio *K*_*S*_/*k*_*cat*_ is not significantly different for the two conditions. Moreover, the increase in glucose transport capacity observed for the 12°C chemostat (1.59) is significantly lower than the decrease in glucose transport capacity caused by the temperature decrease (6.4 fold change). Considering the similar *K*_*S*_/*k*_*cat*_ ratio and the limited difference in *k*_*cat*_ at 30°C for both conditions, the effect must have been small in our experiments.

**Figure 7:**
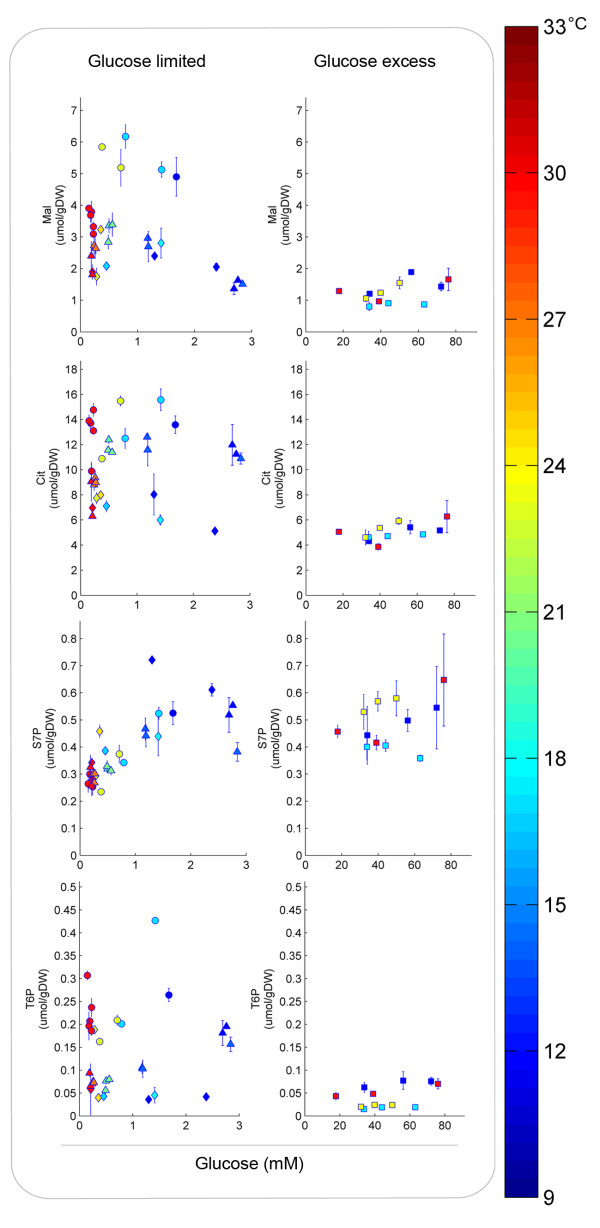
**Intracellular concentrations of metabolites from the tricarboxylic acid pathway, pentose-phosphate pathway and storage carbohydrate metabolism.** Malate (Mal) and citrate (Cit) from the TCA pathway; sedoheptulose-7-phosphate (S7P) from the PPP; trehalose-6-phosphate (T6P) as a precursor of storage carbohydrates. Samples were taken during sinoidal temperature cycles (△), linear temperature shifts from 30°C (○) or 12°C (⋄) and from batches at different temperatures (□). Error bars refer to the standard error of the averages of at least two duplicate samples and two independent experiments. The different colors indicate the temperature of the culture at the time of sampling.

Although this study was focused on yeast glycolysis, pentose phosphate pathway (PPP) metabolites were also found to correlate primarily with extracellular glucose concentration rather than with the temperature. Conversely, tricarboxylic acid pathway (TCA) and storage carbohydrate-related metabolites showed a different behavior, as their levels seemed to be primarily dependent on the initial steady-state temperature (Figure 7). It should be noted that under anaerobic conditions, the TCA, PPP and storage metabolism fluxes are generally much lower than the glycolytic flux [33]. Therefore, changes in metabolites from these pathways will be slower under dynamic conditions. In the SBR experiments no significant difference was observed in the TCA pathway metabolite levels at different temperatures. This was likely due to the higher turnover times (time needed to refill or empty a metabolic pool), rather than different temperature sensitivities. Levels of precursors of storage carbohydrates (e.g. T6P) were too low in glucose-excess conditions to allow a good analysis of the temperature impact in this pathway. However, T6P levels under glucose limited conditions indicate a different regulation mechanism of trehalose-6-phosphate synthase or trehalose-6-phosphate phosphatase compared to glycolytic enzymes. Further model developments and experimental investigations should help to determine if the differences observed for the TCA and storage carbohydrate metabolism are a consequence of transcriptional regulation (reflected, for instance, in different enzyme levels), higher turnover times or significantly different temperature sensitivities. Analyzing the correlation between residual substrate levels and intracellular metabolite concentrations in different temperature conditions might enable to pinpoint which metabolic pathways are the most sensible to temperature perturbations and likely to be relevant in the overall response of *S. cerevisiae* to temperature perturbations.

Evolutionary ‘synchronization’ of the temperature dependencies of the catalytic capacities of enzymes in a metabolic pathway, may confer selective advantages to microorganisms that are subjected to frequent temperature changes. Firstly, changes of metabolite levels in response to temperature changes are minimized, thereby avoiding the need for energetically costly cycles of *de novo* synthesis and degradation of enzymes. This preservation of homeostasis may be particularly important for central metabolism, of which intermediates serve as precursors for several biosynthetic pathways. Secondly, based on metabolic control analysis, it can be inferred that this minimization of changes in metabolite concentrations causes pathway fluxes to be minimally sensitive to temperature variations (see Additional file 4).

Recent studies on temperature as an evolutionary pressure indicate that temperature optima for growth are a key factor for survival of sympatric strains [34-36]. Our results indicate that, in addition to temperature optima, microbial strategies for kinetic adaptation to temperature dynamics (e.g. circadian temperature cycles) may play a key role in determining microbial fitness in natural environments. Further research should reveal whether microorganisms evolved in environments with a steady temperature (e.g. obligate commensals of homoeothermic vertebrates) show a different kinetic regulatory strategy than found for glycolysis in *S. cerevisiae*.

## Conclusions

In view of their size, microorganisms are intrinsically unable to control their temperature. Temperature dynamics in natural environments therefore force microorganisms to continually adapt to temperature changes. In the present study, the glycolytic pathway of *S. cerevisiae* was used as a model to study kinetic regulation strategies of organisms in response to temperature dynamics. By combining both modeling and experimental results we obtained strong indications that the temperature dependencies of the catalytic capacities of yeast glycolytic enzymes are highly similar under *in vivo* conditions. These similarities allow the cells to maintain their homeostasis during circadian temperature cycles, thereby avoiding extensive changes in enzyme levels and minimizing the temperature impact on the growth rate. These results provide new insights in the robustness of yeast central carbon metabolism and open the gate for new studies on temperature as a key parameter for evolution of sympatric strains.

## Methods

### Model description

The published kinetic model for yeast glycolysis [20] was developed to simulate a buffered cell environment where growth is absent and extracellular concentrations are constant. These constraints and the fact that this model was solely based on kinetic parameters that were estimated from *in vitro* experiments, render it unstable in simulations of dynamic conditions. To avoid stability problems when incorporating temperature changes and to better mimic the experimental setups applied in this study, several adjustments were made to the original model.

To enable simulation of scenarios in which extracellular concentrations are highly dynamic, mass balances for glucose, ethanol, glycerol and succinate were included in the model, according to equation (7):

(7) $$\frac{dN_{i}}{dt}=q_{i}N_{X}\left(t\right)+F_{\mathrm{in}}C_{i,in}-F_{\mathrm{out}}C_{i,out}\left(t\right)\text{,}$$

Equation (7) indicates how the amount of component *i* in the broth (*N*_*i*_*(t)*) will change based on its biomass specific production or consumption rate (*q*_*i*_) and the amount of biomass present in the broth (*N*_*X*_*(t)*). The terms *F*_*in*_*C*_*i,in*_ and *F*_*out*_*C*_*i,out*_ refer to transport of the component via, respectively, the inlet and outlet streams of the fermenter. *F* is the flow rate (L.h^-1^) whereas *C*_*i*_ (mM) is the concentration of component *i* in each stream. The parameters were set to be the same as in the experimental setup. The ethanol evaporation rate was taken into account in the mass balance of this compound following the same approach as Cruz *et al.*[13].

Temperature was included in the model by replacing the different catalytic activities by a temperature dependent function. In a previous study, the Ratkowsky model ([37]; equation 8) was found to better describe the temperature impact on metabolic fluxes in anaerobic, glucose-excess cultures of *S. cerevisiae* grown at temperatures between 12 to 30°C than the Arrhenius law [13]. Therefore, the Ratkowsky model was used as the temperature-dependent function *R*_*i*_*(T)* with the parameters obtained by Cruz *et al.*[13].

(8) $$V_{\max,j}\left(T\right)=b_{j}{\left(T-T_{\min}\right)}^{2}$$

At temperatures between 12 and 30°C, temperature has little impact on the yields of biomass and fermentation products in anaerobic *S. cerevisiae* cultures [13]. Therefore the stoichiometric coefficient *b*_*j*_ for each reaction was calculated from the ratio of its flux and the glucose uptake rate (*v*_*consumption*_) in the original conditions of the Teusink model (30°C):

(9) $$b_{j}=b_{\mathrm{Glc}}\frac{v_{j}^{30{}^{\circ}C}}{v_{\mathrm{consumption}}^{30{}^{\circ}C}}$$

The model was implemented and run in gPROMS (Process Systems Enterprise).

### Strain and growth conditions

*Saccharomyces cerevisiae* CEN.PK113-7D Mat**a**[38,39] was grown anaerobically in medium containing 0.3 g.L^-1^ of (NH_4_)_2_SO_4_, 0.3 g.L^-1^ of K_2_H_2_PO_4_, 3.0 g.L^-1^ NH_4_H_2_PO_4_, 0.5 g.L^-1^ MgSO_4_.7H_2_O, 0.4 g.L ^-1^ of Tween 80, 10 mg.L^-1^ ergosterol and glucose (25 g.L^-1^). The medium was supplemented with 1 ml.L^-1^ each of a trace element solution and a vitamin solution [40] as well as with 0.15 g.L^-1^ of antifoam (Silcolapse 5020, Bluestar Silicones, St. Fons, France).

The sequential batch experiments were performed in 1L fermenters with a working volume of 750 ml, whereas 2L fermenters were used for chemostat cultivation (Applikon, Schiedam, The Netherlands). The working volumes for linear-temperature-shifts (LTS) and circadian-temperature-cycles (CTC) experiments were 1.0 and 1.4 L, respectively. The stirring speed was set at 600 rpm for all cultures and the pH was controlled to 5.0 through automatic addition of 2.0 M KOH or 2.0M of H_2_SO_4_ using a Biostat Bplus controller (Sartorius BBI Systems, Melsungen, Germany). The impact of temperature on the pH measurement was taken into account by sensor calibration. In order to avoid oxygen diffusion into the cultures, Norprene tubing was used for all connections and both medium vessel and fermenter were continuously sparged with pure nitrogen gas (N_2_) at a flow rate of 0.35 vvm via an Ion Science Saga digital flow meter (Cambridge, UK).

An overview of the different temperature studies made is given in Figure 1. For the experiments in glucose excess conditions (SBR), two sequential batch cycles were run at each temperature (30, 24, 18 and 12°C) before samples were taken in the third cycle, to assure that cells were fully adapted to the new temperature [13]. Duplicate samples were taken at three different time points of the exponential phase for each temperature.

The linear-temperature-shifts (LTS) and circadian-temperature-cycles (CTC) experiments were performed in glucose-limited cultures grown at a dilution rate of 0.03 h^-1^. After 5 residence times at constant temperature (30 or 12°C), the temperature in LTS experiments was linearly increased or decreased at a rate of 0.2°C.min^-1^. Samples were taken when temperature reached 30, 24, 18 and 12°C and up to 3 h after the temperature returned to the initial set-point. The temperature profiles in the SBR and LTS experiments were defined and controlled via the MFCS/win 2.1 software (Sartorius BBI Systems, Melsungen, Germany).

For CTC-experiments, a pre-programmed sinoid temperature profile (temperature (°C) = 21+9sin(π/12·t (h) +1.57) was started after 3 residence times at a constant temperature of 30°C. This profile was designed to mimic a circadian temperature cycle. Low-temperature thermostats (Lauda RE304, Lauda-Königshofen, Germany) ensured that the temperature was precisely controlled throughout the experiments. Samples were taken during the 5^th^ and the 6^th^ temperature cycle, by which time carbon dioxide profiles and metabolite concentrations during consecutive cycles were highly similar. To minimize disturbance, sampling volumes did not exceed 5% of the reactor volume during a single temperature cycle and minimum intervals of 3 h were maintained between sampling points.

### Analytical methods

Extracellular glucose was measured in 2 ml of broth samples, rapidly taken with syringes containing steel beads at −20°C [41]. The number of beads was adjusted for each initial broth temperature, such that the temperature of all samples would decrease instantaneously to 1°C. Residual glucose concentrations were measured via high-performance liquid chromatography with a Bio-Rad Aminex HPX-87H column at 60°C. The column was eluted with 5 mM phosphoric acid at a flow rate of 0.6 ml.min^–1^. Glucose was detected with a Waters 2410 refractive index detector. Biomass dry weight was measured in duplicate samples as described in [26].

Intracellular metabolite samples were taken by withdrawing 1.2 ml of broth directly to 6 ml of 100% methanol at −40°C via a rapid sampling setup. Samples were washed with cold methanol and extracted with boiling ethanol as described in [41]. The concentrations of glucose-6-phosphate (G6P), fructose-6-phosphate (F6P), fructose-1,6-bisphosphate (FBP), phospho-*enol*-pyruvate (PEP), glycerol-3-phosphate (G3P), malate (MAL), fumarate (FUM), succinate (SUC), α-ketoglutarate (αKG), citrate (CIT), glucose-1-phosphate (G1P), UDP-glucose (UDPgluc), trehalose-6-phosphate (T6P), mannose-6-phosphate (M6P), 6-phosphogluconate (P6G) and sedoheptulose-7-phosphate (S7P) were measured by LC-MS according to the protocol developed by [42]. The nucleotide concentrations (ATP, ADP and AMP) were measured according to [43]. Uniformly labeled ^13^C-cell-extract was applied in both analytical platforms as internal standard [44].

*In vitro* enzyme assays of the glycolytic enzymes were performed with freshly prepared cell extracts on a Hitachi model 100–60 spectrophotometer at 30°C and 340 nm (ε_340_ of reduced pyridine-dinucleotide cofactors 6.3 mM^-1^). All enzymes were assayed as described previously [45], with the exception of phosphofructokinase (PFK; EC 2.7.1.11), which was assayed according to [46], with minor modifications. The assay mixture contained: imidazole/HCl (pH 7.0) 50 mM, MgCl_2_ 5 mM, NADH 0.15 mM, fructose 2,6-diphosphate 0.10 mM, aldolase (EC 4.1.2.13) (Sigma) 2.1 U ml^-1^, α-glycerophosphate dehydrogenase-triosephosphate isomerase, 1.2 U ml^-1^ and 12.4 U ml^-1^, respectively (Sigma) and cell extract. After recording background activity with 0.5 mM fructose 6-phosphate, the reaction was started with 1.0 mM ATP. All assays were performed at two concentrations of cell extract. Specific activities in duplicate experiments differed by less than 12%. Enzyme activities are expressed as μmol substrate converted per min per mg protein [U (mg protein)^-1^. Protein concentrations in cell extracts were determined according to [47], using dried bovine serum albumin (fatty-acid free; Sigma) as the standard.

## Competing interests

The authors declare that they have no competing interests.

## Authors’ contributions

ALC, MH, PDL, JP, JH and WvG designed the research. ALC performed the sequential batch and linear-temperature-shift experiments. ALC and SAW performed the mathematical modeling. MH and GHD conducted the circadian cycle experiments. MH performed the enzyme activity assays. ALC and MH wrote the manuscript with substantial input from the other authors. All authors read and approved the final manuscript.

## Supplementary Material

Additional file 1 **Figure S1.** A. Intracellular concentrations derived from model simulations considering that *k*_*cat*_ of phosphofructokinase (PFK) is 2 times less sensitive to temperature than the other glycolytic enzymes. The symbols refer to simulations of: sinoidal temperature cycles (▵) and linear temperature shifts from 30°C steady-state chemostats (○); batch fermentations at different temperatures (□). The colors indicate the culture temperature at the time of sampling. All concentrations are normalized to the levels under glucose excess conditions at 30°C. B. Intracellular concentrations derived from model simulations considering that *k*_*cat*_ of pyruvate kinase (PYK) is 2 times less sensitive to temperature than the other glycolytic enzymes. The symbols refer to simulations of: sinoidal temperature cycles (▵) and linear temperature shifts from 30°C steady-state chemostats (○); batch fermentations at different temperatures (□). The colors indicate the culture temperature at the time of sampling. All concentrations are normalized to the levels under glucose excess conditions at 30°C. Click here for file

Additional file 2 **Figure S2.** Enzymatic capacities (Vmax) of the glycolytic enzymes that are not shown in figure 3, estimated from in vitro enzyme activity assays measured at 30°C in cell free extracts of S.cerevisiae cultivated in glucose-limited anaerobic chemostats subjected to circadian temperature cycles (CTC). A. Hexokinase (HXK); B. Fructose bi-phosphate aldolase (FBA); C. Triose phosphate isomerase (TPI); D. Phosphoglycerate kinase (PGK); E. Phosphoglycerate mutase (PGM); F. Enolase (ENO). Click here for file

Additional file 3 **Figure S3.** Nucleotide levels profiles as a function of the extracellular glucose from experiments with sinoidal temperature cycles (▵), linear temperature shifts from 30°C (○) or 12°C (X) and from batches at different temperatures (□). The error bars refer to the standard error of two duplicate samples from at least two independent runs of experiments. The different colors indicate the temperature of the sample. Click here for file

Additional file 4 Example of minimal flux changes upon temperature perturbations. Click here for file
