# Supplementary material for: Similar temperature dependencies of glycolytic enzymes: an evolutionary adaptation to temperature dynamics?
Source: BMC Syst Biol. 2012 Dec 7;6:151. doi: 10.1186/1752-0509-6-151 (PMC3554419; doi:10.1186/1752-0509-6-151)
Supplement: Additional file 1 — Figure S1. A. Intracellular concentrations derived from model simulations considering that kcat of phosphofructokinase (PFK) is 2 times less sensitive to temperature than the other glycolytic enzymes. The symbols refer to simulations of: sinoidal temperature cycles (▵) and linear temperature shifts from 30°C steady-state chemostats (○); batch fermentations at different temperatures (□). The colors indicate the culture temperature at the time of sampling. All concentrations are normalized to the levels under glucose excess conditions at 30°C. B. Intracellular concentrations derived from model simulations considering that kcat of pyruvate kinase (PYK) is 2 times less sensitive to temperature than the other glycolytic enzymes. The symbols refer to simulations of: sinoidal temperature cycles (▵) and linear temperature shifts from 30°C steady-state chemostats (○); batch fermentations at different temperatures (□). The colors indicate the culture temperature at the time of sampling. All concentrations are normalized to the levels under glucose excess conditions at 30°C. [file 1752-0509-6-151-S1.pdf]

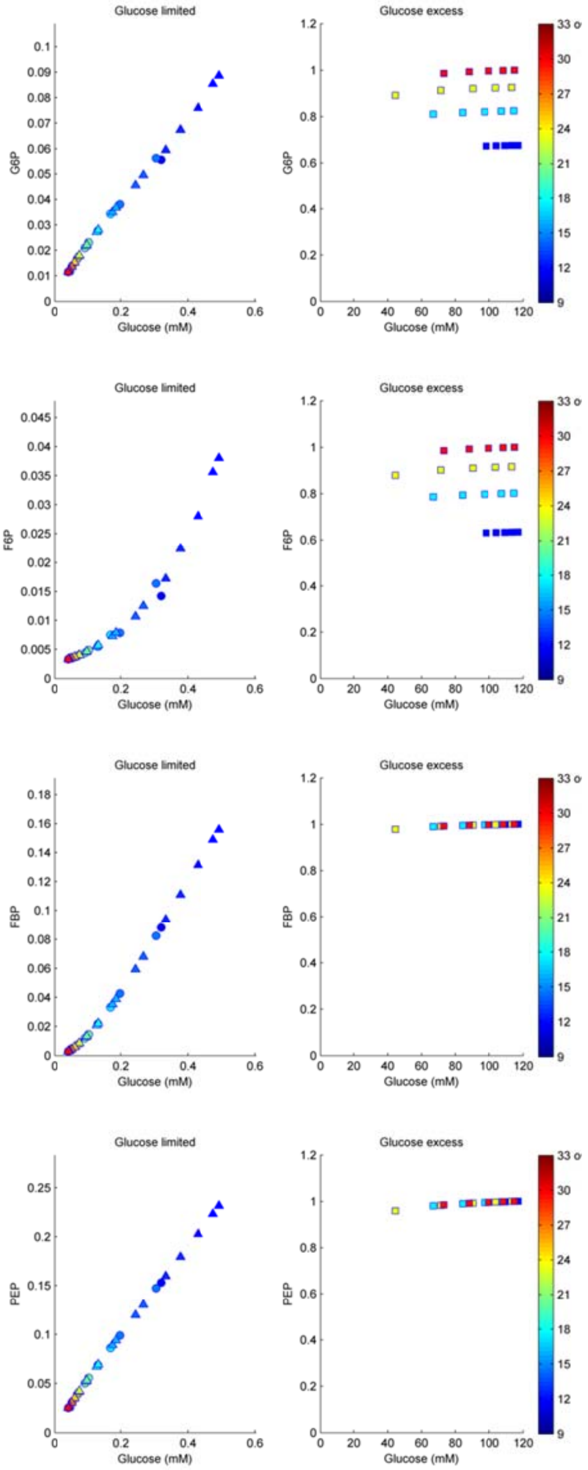

Additional Figure 1A - Intracellular concentrations derived from model simulations considering that k_{cat} of phosphofructokinase (PFK) is 2 times less sensitive to temperature than the other glycolytic enzymes. The symbols refer to simulations of: sinoidal temperature cycles (Δ) and linear temperature shifts from 30 °C steady-state chemostats (○); batch fermentations at different temperatures (□). The colors indicate the culture temperature at the time of sampling. All concentrations are normalized to the levels under glucose excess conditions at 30 ^{o}C.

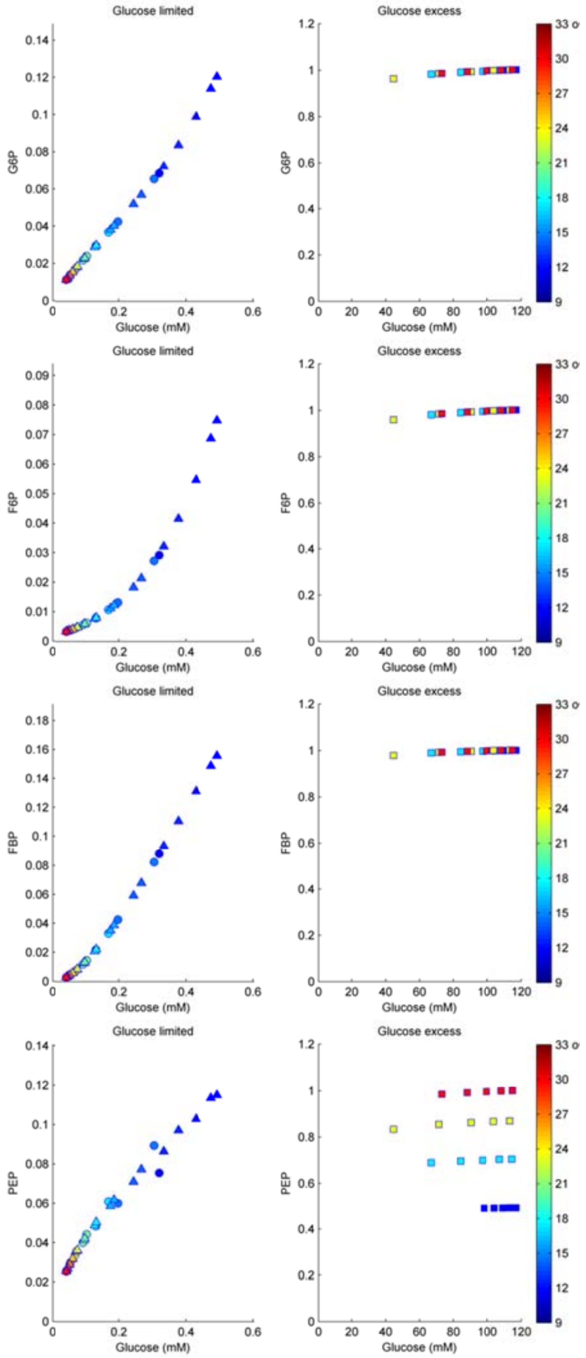

Additional Figure 1B - Intracellular concentrations derived from model simulations considering that k_{cat} of pyruvate kinase (PYK) is 2 times less sensitive to temperature than the other glycolytic enzymes. The symbols refer to simulations of: sinoidal temperature cycles (Δ) and linear temperature shifts from 30 °C steady-state chemostats (○); batch fermentations at different temperatures (□). The colors indicate the culture temperature at the time of sampling. All concentrations are normalized to the levels under glucose excess conditions at 30 ^{o}C.
