# Supplementary material for: Similar temperature dependencies of glycolytic enzymes: an evolutionary adaptation to temperature dynamics?
Source: BMC Syst Biol. 2012 Dec 7;6:151. doi: 10.1186/1752-0509-6-151 (PMC3554419; doi:10.1186/1752-0509-6-151)
Supplement: Additional file 2 — Figure S2. Enzymatic capacities (Vmax) of the glycolytic enzymes that are not shown in figure 3, estimated from in vitro enzyme activity assays measured at 30°C in cell free extracts of S.cerevisiae cultivated in glucose-limited anaerobic chemostats subjected to circadian temperature cycles (CTC). A. Hexokinase (HXK); B. Fructose bi-phosphate aldolase (FBA); C. Triose phosphate isomerase (TPI); D. Phosphoglycerate kinase (PGK); E. Phosphoglycerate mutase (PGM); F. Enolase (ENO). [file 1752-0509-6-151-S2.pdf]

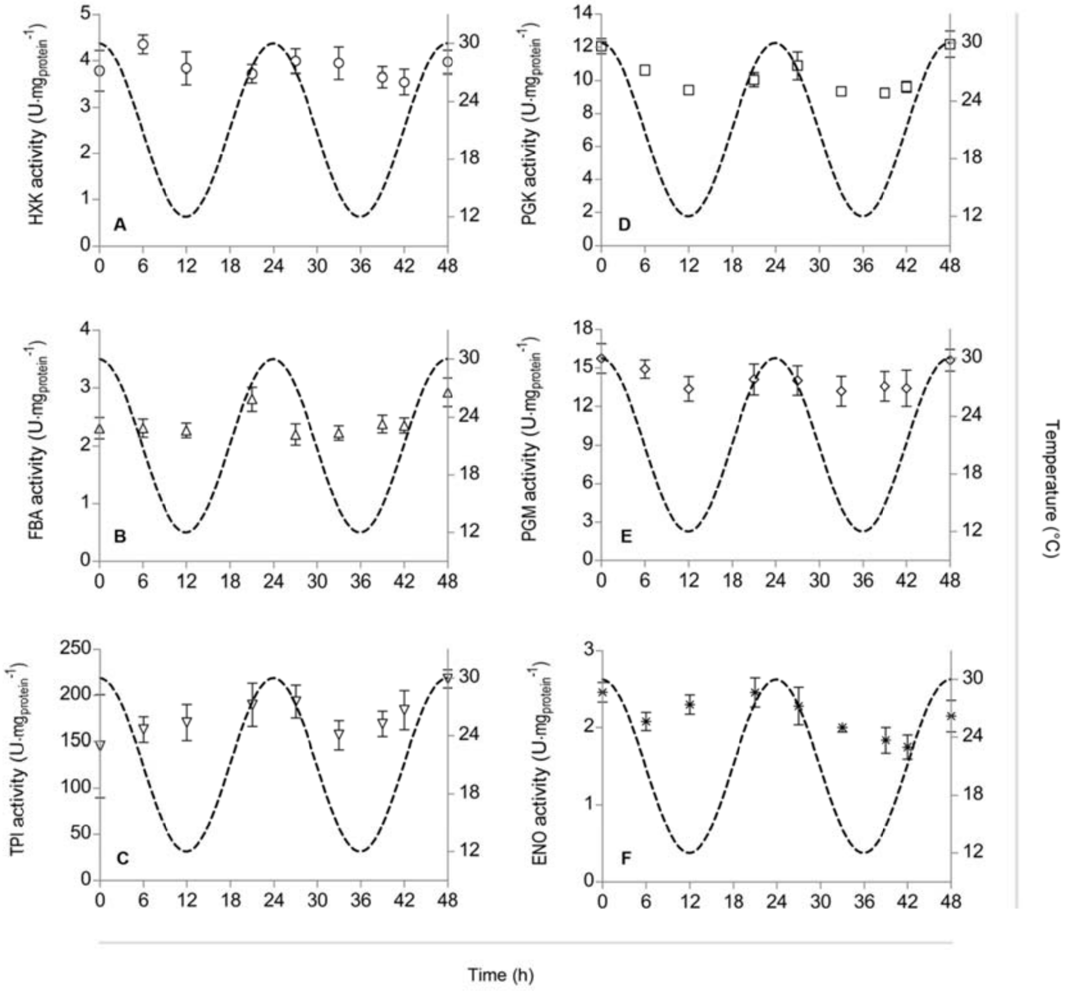

Additional Figure 2 - Enzymatic capacities (V_{max}) of the glycolytic enzymes that are not shown in figure 3, estimated from *in vitro* enzyme activity assays measured at 30 °C in cell free extracts of *S.cerevisiae* cultivated in glucose-limited anaerobic chemostats subjected to circadian temperature cycles (CTC). A. Hexokinase (HXK); B. Fructose bi-phosphate aldolase (FBA); C. Triose phosphate isomerase (TPI); D. Phosphoglycerate kinase (PGK); E. Phosphoglycerate mutase (PGM); F. Enolase (ENO).
