# Supplementary material for: Similar temperature dependencies of glycolytic enzymes: an evolutionary adaptation to temperature dynamics?
Source: BMC Syst Biol. 2012 Dec 7;6:151. doi: 10.1186/1752-0509-6-151 (PMC3554419; doi:10.1186/1752-0509-6-151)
Supplement: Additional file 3 — Figure S3. Nucleotide levels profiles as a function of the extracellular glucose from experiments with sinoidal temperature cycles (▵), linear temperature shifts from 30°C (○) or 12°C (X) and from batches at different temperatures (□). The error bars refer to the standard error of two duplicate samples from at least two independent runs of experiments. The different colors indicate the temperature of the sample. [file 1752-0509-6-151-S3.pdf]

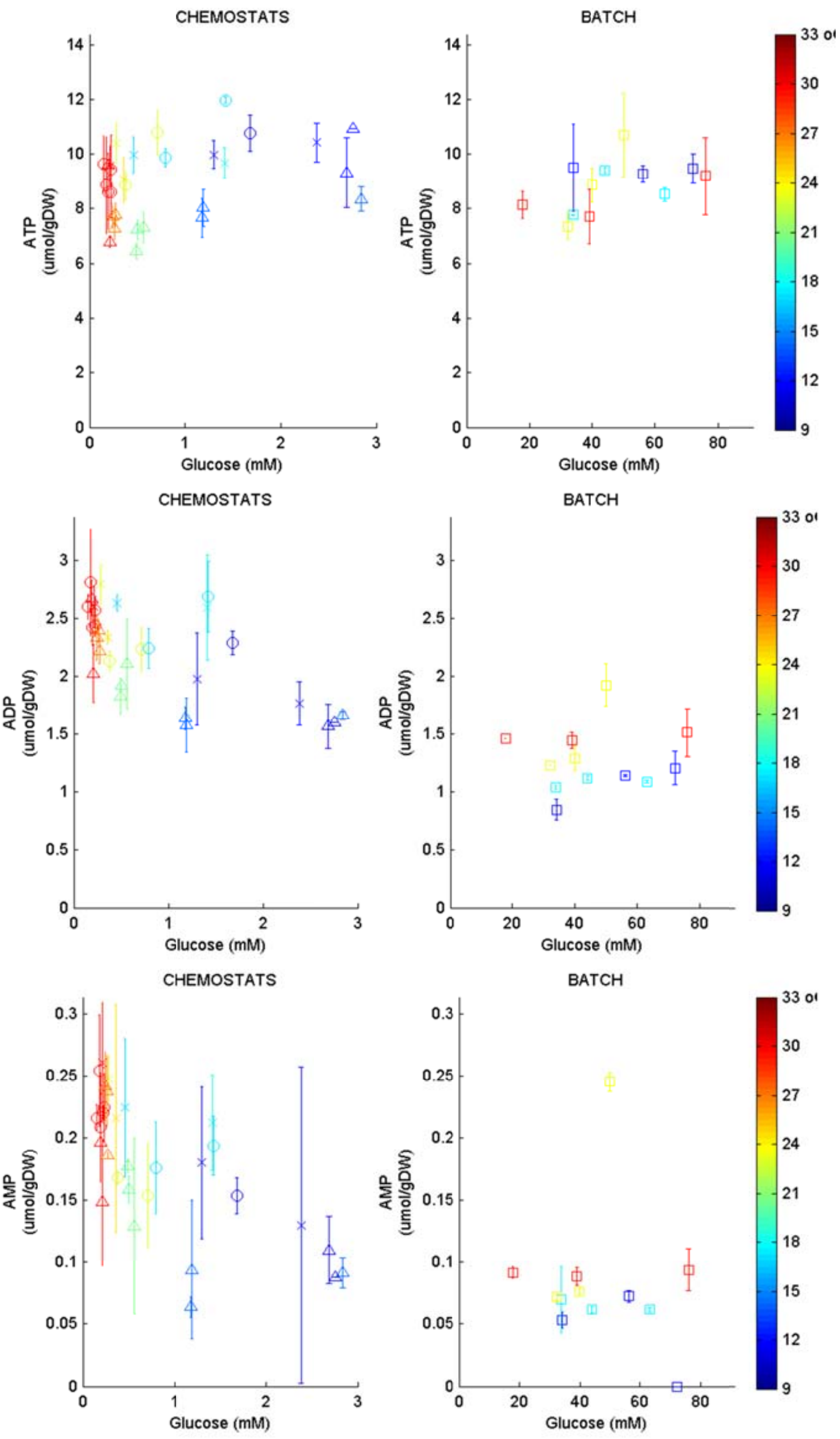

Additional Figure 3 - Nucleotide levels profiles as a function of the extracellular glucose from experiments with sinoidal temperature cycles (Δ), linear temperature shifts from 30 °C (O) or 12 °C (X) and from batches at different temperatures (☐). The error bars refer to the standard error of two duplicate samples from at least two independent runs of experiments. The different colors indicate the temperature of the sample.
