# Supplementary material for: Similar temperature dependencies of glycolytic enzymes: an evolutionary adaptation to temperature dynamics?
Source: BMC Syst Biol. 2012 Dec 7;6:151. doi: 10.1186/1752-0509-6-151 (PMC3554419; doi:10.1186/1752-0509-6-151)
Supplement: Additional file 4 — Example of minimal flux changes upon temperature perturbations. [file 1752-0509-6-151-S4.pdf]

Demonstration that similar temperature-dependencies of k_{cat} lead to minimal flux change upon temperature perturbations

To demonstrate how the temperature dependency of k_{cat} influences the flux change, we will consider once more the simple network (3) presented in the main text. According to metabolic control analysis (Van Gulik *et al*, 2003), the change in flux through the pathway (J) compared to the flux at a reference condition (with flux J^0 and biomass specific capacity V_i^0) can be quantified based on the change in enzymatic capacity of the three different enzymes (V_i / V_i^0) and the flux control coefficients $C^{J_i^0}$, in such way that $\sum_{i=1}^{3} C^{J_i^0} = 1$.

$$\frac{J}{J^0} = \frac{1}{C^{J_1^0}\frac{V_1^0}{V_1} + C^{J_2^0}\frac{V_2^0}{V_2} + C^{J_3^0}\frac{V_3^0}{V_3}} \qquad (1)$$

As we have discussed in the main section, the enzyme levels are not affected by temperature, meaning that the change in V_i^0 / V_i will be uniquely a consequence of the temperature impact on the catalytic activity (k_{cat}) according once more to function $R_i(T)$.

$$\frac{V_i^0}{V_i} = \frac{k_{cat\,i}^{\;0}}{k_{cat\,i}} = \frac{1}{R_i(T)} \qquad (2)$$

From equation (2) and knowing that for temperatures below the optimal growth temperature (this study) k_{cat} decreases with decreasing temperature, it can be seen that:

$$\begin{cases} T < T^0 \rightarrow 0 < R_i(T) < 1 \\ T = T^0 \rightarrow R_i(T) = 1 \\ T > T^0 \rightarrow R_i(T) > 1 \end{cases} \qquad (3)$$

By replacing equation (2) in (1) we obtain the relation between the change in flux with temperature:

$$\frac{J}{J^0} = \frac{1}{\frac{C^{J_1^0}}{R_1(T)} + \frac{C^{J_2^0}}{R_2(T)} + \frac{C^{J_3^0}}{R_3(T)}} \quad (4)$$

We will now consider that, for temperatures lower than the reference temperature (T^0), the catalytic activity of enzyme 1 decreases less severely than for enzymes 2 and 3. This means that $R_1(T)$ will be higher than $R_2(T)$ or $R_3(T)$. Multiplying equation (4) by $R_1(T)$ we obtain equation (4) where the ratios $R_1(T)/R_2(T)$ and $R_1(T)/R_3(T)$ will always be higher than 1.

$$\frac{J}{J^0} = \frac{R_1(T)}{C^{J_1^0} + C^{J_2^0}\frac{R_1(T)}{R_2(T)} + C^{J_3^0}\frac{R_1(T)}{R_3(T)}} \quad (5)$$

If, on the other hand, we speculate that evolution led to a situation where all k_{cat} have the same low-temperature-sensitivity, all enzymes would have the same temperature function as the one of enzyme 1 ($R_1(T) = R_2(T) = R_3(T)$). This means that, when multiplying equation (4) by $R_1(T)$ equation (6) can be derived.

$$\frac{J}{J^0} = \frac{R_1(T)}{C^{J_1^0} + C^{J_2^0} + C^{J_3^0}} = R_1(T) \quad (6)$$

Because the denominator in equation (5) will always be higher than 1, the flux drecrase as a consequence of temperature drop is minimal only if k_{cat} values of the different enzymes have the same temperature dependency.

Even in cases where J/J^0 is different than $R_1(T)$ (for instance if the overall change in flux is the average of the functions $R_1(T)$, $R_2(T)$ and $R_3(T)$), only in the extreme cases where the enzyme with the highest temperature sensitivity has no impact on the flux through the pathway ($C^{J_i^0}$ close to 0), will the flux change be less than in the situation where all enzymes have similar temperature sensitivities (Figure 1).

This points out that, besides minimizing changes in intracellular metabolite levels, identical temperature sensitivities of the enzymes in a pathway can also minimize the changes in flux in response to temperature fluctuations. This can be beneficial in competitive environments since the growth rate of the microorganisms would not be drastically reduced when temperature decreases.

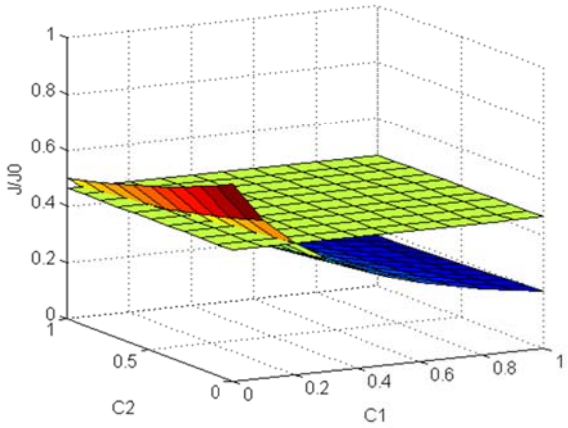

Figure 1: Impact of the control coefficients $C_i^{J^0}$ in the overall change in flux with temperature. For the simulation considering different temperature sensitivities of the different enzymes it was assumed that the function $R_i(T)$ was 0.7, 0.5 and 0.2 for, respectively, enzymes 1, 2 and 3. The green surface represents the situation where all the enzymes have the same temperature dependencies, considering that the overall change in flux J/J^0 would be the average of the $R_i(T)$ values used in the previous simulation.

References

Van Gulik W, Van Winden WA, Heijnen JJ (2003) Flux analysis: modeling and engineering solutions. In *Handbook of Industrial Cell Culture: Mammalian, Microbial, and Plant Cells*, Vinci VA, Parekh SR (eds), pp 349-392. New Jersey, USA: Humana Press Inc.
